# Supplementary material for: Splice-Junction-Based Mapping of Alternative Isoforms in the Human Proteome
Source: Cell Rep. Author manuscript; Available in PMC 2020 Jan 15. (PMC6961840; doi:10.1016/j.celrep.2019.11.026)
Supplement: 2 [file NIHMS1546469-supplement-2.zip › DF1/PXD009737/TestisTrypsin/TestisTrypsin_4_CLTCL1_LQEHFQLQNLGINPANIGFSTLTMESDK.pdf]

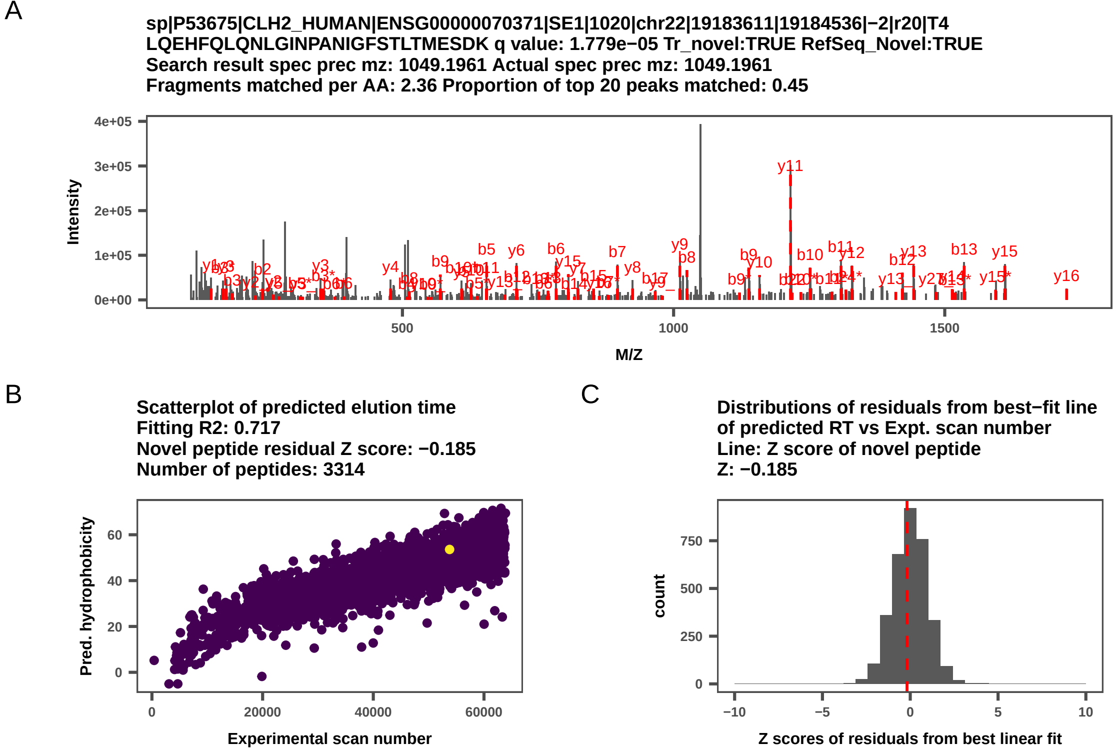
A
sp|P53675|CLH2_HUMAN|ENSG00000070371|SE1|1020|chr22|19183611|19184536|−2|r20|T4
LQEHFQLQNLGINPANIGFSTLTMESDK q value: 1.779e−05 Tr_novel:TRUE RefSeq_Novel:TRUE
Search result spec prec mz: 1049.1961 Actual spec prec mz: 1049.1961
Fragments matched per AA: 2.36 Proportion of top 20 peaks matched: 0.45
Intensity
4e+05
3e+05
2e+05
1e+05
0e+00
M/Z
500
1000
1500
y11
b5
y6
b6
b7
y8
y9
b8
b9
y10
b10
b11
y12
b12
y13
b13
y15
y15*
y16
y4
y3
b2
B
Scatterplot of predicted elution time
Fitting R2: 0.717
Novel peptide residual Z score: −0.185
Number of peptides: 3314
Pred. hydrophobicity
Experimental scan number
0
20
40
60
20000
40000
60000
C
Distributions of residuals from best−fit line
of predicted RT vs Expt. scan number
Line: Z score of novel peptide
Z: −0.185
count
0
250
500
750
Z scores of residuals from best linear fit
−10
−5
0
5
10
